# Anisotropic Spin Fluctuations Induced by Spin‐Orbit Coupling in a Misfit Layer Compound (LaSe)_1.14_(NbSe_2_)

**DOI:** 10.1002/advs.202403824

**Published:** 2024-08-29

**Authors:** Min Shan, Shunjiao Li, Ye Yang, Dan Zhao, Jian Li, Linpeng Nie, Zhimian Wu, Yanbing Zhou, Lixuan Zheng, Baolei Kang, Tao Wu, Xianhui Chen

**Affiliations:** ^1^ Hefei National Research Center for Physical Sciences at the Microscale University of Science and Technology of China Hefei Anhui 230026 China; ^2^ CAS Key Laboratory of Strongly‐coupled Quantum Matter Physics Department of Physics University of Science and Technology of China Hefei Anhui 230026 China; ^3^ Collaborative Innovation Center of Advanced Microstructures Nanjing University Nanjing 210093 China; ^4^ Hefei National Laboratory University of Science and Technology of China Hefei 230088 China

**Keywords:** 2D transition metal dichalcogenides, misfit layer compounds, spin fluctuations, spin‐orbit coupling, weak antilocalization effect

## Abstract

Spin‐orbit coupling (SOC) has significant effects on the superconductivity and magnetism of transition metal dichalcogenides (TMDs) at the 2D limit. Although 2D TMD samples possess many exotic properties different from those of bulk samples, experimental characterization in this field is still limited, especially for magnetism. Recent studies have revealed that bulk misfit layer compounds (MLCs) with (LaSe)_1.14_(NbSe_2_)_n = 1,2_ exhibit an Ising superconductivity similar to that of heavily electron‐doped NbSe_2_ monolayers. This offers an opportunity to study the effect of SOC on the magnetism of 2D TMDs. Here, the possible SOC effect in (LaSe)_1.14_(NbSe_2_) is investigated by measuring nuclear magnetic resonance (NMR) and electrical transport. It is found that the LaSe layer not only functions as a charge reservoir for transferring electrons into the NbSe_2_ layer but also remarkably influences the local electronic environment around the ^93^Nb nuclei. More importantly, the significant SOC induces both a weak antilocalization (WAL) effect and anisotropic spin fluctuations in noncentrosymmetric NbSe_2_ layers. The present work contributes to a deep understanding of the role of the SOC effect in 2D TMDs and supports MCLs as an intriguing platform for exploring exotic physical properties within the 2D limit.

## Graphical abstract

In misfit layer compound (LaSe)_1.14_(NbSe_2_), LaSe layers effectively reduce the dimensionality of NbSe_2_ layers, which makes spin‐orbit coupling become apparent. The weak antilocalization effect and spin fluctuations caused by spin‐orbit coupling are detected through transport and NMR experiments. This study proves that MCLs are a suitable platform for exploring exotic physical properties of transition metal dichalcogenides within the 2D limit.

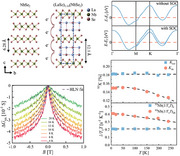

## 1 Introduction

**Figure 1:**
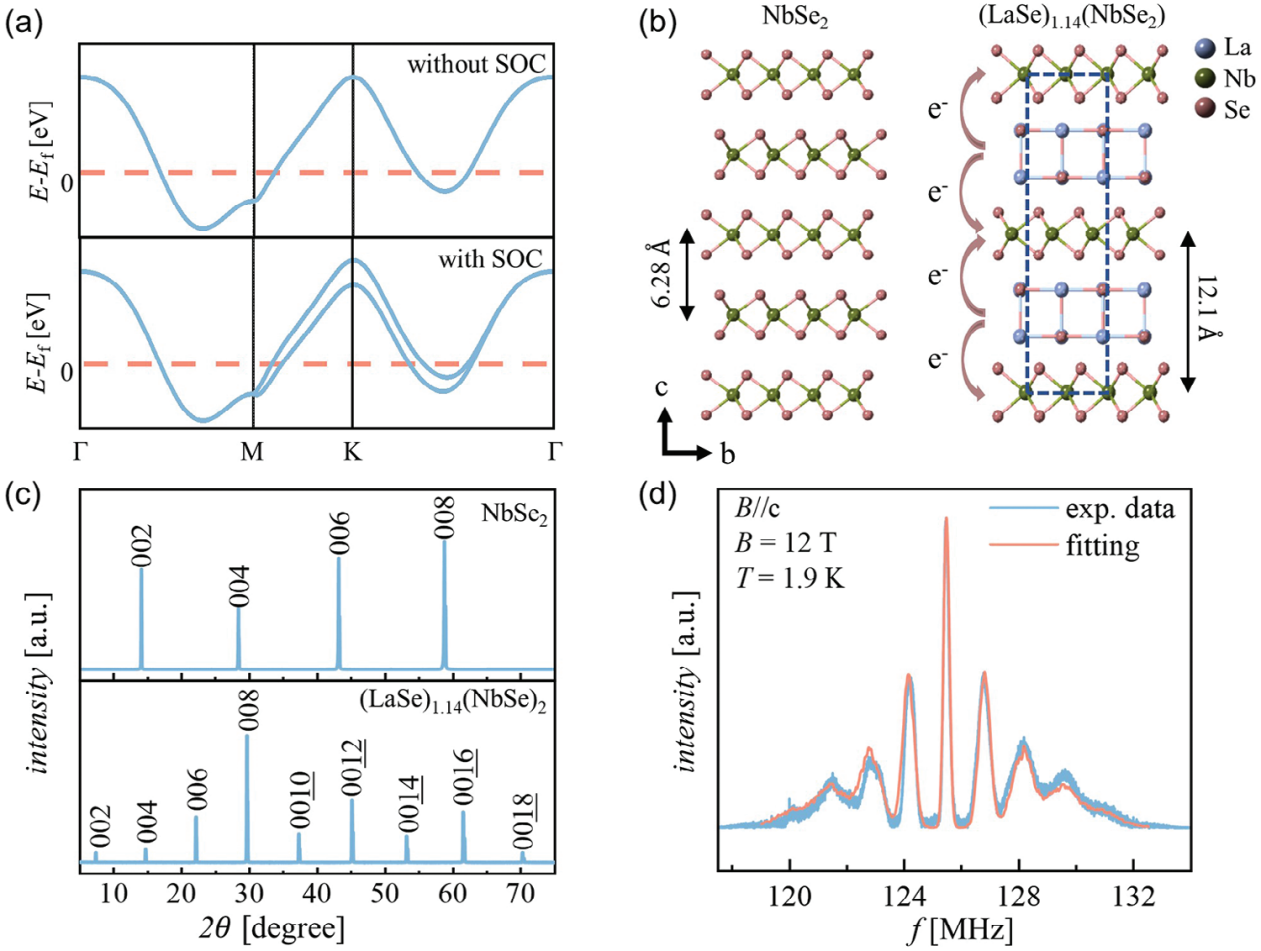
SOC effects on the NbSe_2_ monolayer and structural characterization of bulk 2H‐NbSe_2_ and (LaSe)_1.14_(NbSe_2_). a) The band structure of the NbSe_2_ monolayer without/with SOC. b) Schematic diagram of the crystal structure of bulk 2H‐NbSe_2_ and (LaSe)_1.14_(NbSe_2_). c) XRD patterns of bulk 2H‐NbSe_2_ and (LaSe)_1.14_(NbSe_2_). d) The ^93^Nb NMR spectrum of (LaSe)_1.14_(NbSe_2_) and the related fitting result.

SOC profoundly impacts the physical properties of TMDs, giving rise to intriguing physical phenomena, such as anisotropic magnetism and Ising superconductivity.^[^ ^1^, ^2^, ^3^, ^4^ ^]^ As one of the canonical TMDs, 2H‐NbSe_2_ has undergone extensive scrutiny through both experimental and theoretical investigations. In bulk 2H‐NbSe_2_, SOC induces an anisotropic effective *g* factor, which leads to anisotropic spin susceptibility.^[^ ^1^, ^5^ ^]^ As the dimensionality decreases, the role of the SOC becomes more pivotal. For instance, the manifestation of Ising superconductivity in an NbSe_2_ monolayer is attributed to SOC effects.^[^ ^3^, ^4^ ^]^ Under the protection of out‐of‐plane mirror symmetry, SOC and the lack of inversion symmetry not only lead to the band splitting at the K (K') points (see **Figure** **1**a) but also make the spins locked along the off‐plane direction. This peculiar spin‐orbit interaction results in a novel superconductivity with an in‐plane critical field beyond the Pauli limit.^[^ ^3^ ^]^ Additionally, theoretical calculations have also predicted that SOC can produce anisotropic spin fluctuations in an NbSe_2_ monolayer.^[^ ^6^ ^]^ Whether long‐range magnetic order or spin fluctuations coexist with superconductivity can dramatically affect the fundamental properties of superconductivity in an NbSe_2_ monolayer. Theoretically, the interaction between the momentum‐dependent phonon and spin fluctuations could produce mixed parity of order parameters in Ising superconductivity.^[^ ^7^ ^]^ However, theoretical calculations regarding the magnetism of NbSe_2_ monolayers have yielded conflicting results. Some calculation results indicate that the NbSe_2_ monolayer is a nonmagnetic system,^[^ ^6^, ^7^, ^8^, ^9^ ^]^ while others indicate that the magnetic ground state of the NbSe_2_ monolayer is ferromagnetic or antiferromagnetic.^[^ ^10^, ^11^, ^12^ ^]^ As far as we know, due to the difficulty of measuring the intrinsic susceptibility or magnetic fluctuations in 2D samples, direct experimental evidence is still lacking.

To solve this challenge of measuring the intrinsic magnetism of 2D TMDs, several novel experimental approaches have been proposed. For example, increasing the distance between adjacent layers in 3D bulk materials could reduce interlayer interactions to achieve a 2D electronic state.^[^ ^13^, ^14^, ^15^ ^]^ The intercalation of organic cations into bulk 2H‐NbSe_2_ provides an effective way to achieve a 2D electronic structure and yield environmentally stable Ising superconductivity.^[^ ^13^ ^]^ The superconducting temperature (*T*_c_) of the intercalated bulk is significantly higher than that of the NbSe_2_ monolayer.^[^ ^13^ ^]^ Similarly, a class of 3D van der Waals heterostructures can also achieve an effective 2D electronic state of TMDs, where the TMD (TX_2_) block is separated by a chalcogenide (MX) block with a NaCl‐type structure.^[^ ^16^, ^17^, ^18^, ^19^, ^20^ ^]^ The TX_2_ block contains one to three layers of TMDs, while the MX block contains only a single layer of chalcogenides. The lattice structures and symmetries of the two blocks are different, resulting in a lattice misfit along the *a*‐axis. Hence, the compounds are named MLCs. Since the lack of commensurability hinders the formation of interlayer chemical bonds, most MLCs exhibit van der Waals interactions between adjacent blocks. The difference in chemical potential between the two blocks prompts a large amount of charge transfer from the MX blocks to the TX_2_ blocks, which could stabilize the lattice structure. Many experiments have demonstrated that the physical properties of MLCs are mainly determined by TMD blocks.^[^ ^16^, ^17^, ^18^, ^19^, ^20^ ^]^ Specifically, in (LaSe)_1.14_(NbSe_2_), the superconducting state exhibits Ising protection with an in‐plane critical field of ≈16 T, which is well above the Pauli limit.^[^ ^20^ ^]^ Angle‐resolved photoemission spectroscopy (ARPES) and density functional theory (DFT) calculations show that the electronic structure of (LaSe)_1.14_(NbSe_2_) is similar to that of the heavily electron‐doped NbSe_2_ monolayer.^[^ ^20^ ^]^

In this work, we systematically investigated the physical properties of (LaSe)_1.14_(NbSe_2_) by NMR and transport experiments. Our findings reveal that LaSe layers not only protect the 2D properties of NbSe_2_ layers but also significantly affect the chemical environment of the NbSe_2_ layers. Additionally, the reduction in dimensionality accentuated the manifestation of SOC effects in (LaSe)_1.14_(NbSe_2_). We not only detected the quasi‐2D WAL effect arising from strong SOC but also found the anisotropic spin fluctuations induced by SOC in noncentrosymmetric NbSe_2_ layers.

## 2 Results and Discussion

### 2.1 Structural Characterization

To date, research on the crystal structure of MCLs has mainly focused on periodic lattices in different blocks, and few studies have been conducted on lattice misfits between different blocks.^[^ ^16^, ^17^, ^19^ ^]^ However, to fully understand the structural features of MCLs, we need to consider the influence of the incommensurability between different blocks on the lattice structure. As a probe that is sensitive to the local structural environment, NMR spectroscopy can help us to better understand the above misfit structure. Here, the structure of (LaSe)_1.14_(NbSe_2_) was characterized by both X‐ray diffraction (XRD) and NMR experiments. X‐ray spectroscopy was used to probe the periodic structure of the crystal, while NMR was used to detect the local environment around ^93^Nb nuclei with a nuclear spin number (*I*) of 9/2. Figure 1b shows a comparison of the lattice structures of bulk 2H‐NbSe_2_ and (LaSe)_1.14_(NbSe_2_). The XRD patterns of bulk 2H‐NbSe_2_ and (LaSe)_1.14_(NbSe_2_) are also shown in Figure 1c. According to the Bragg equation, the distance between adjacent NbSe_2_ layers in (LaSe)_1.14_(NbSe_2_) increases from 6.28 Å in bulk 2H‐NbSe_2_ to 12.1 Å. In ^93^Nb NMR experiments, due to the quadrupole effect from the interaction between the electric quadrupole moment of nuclei and the electric field gradient (EFG), the single NMR spectrum is split into nine transition lines with the same interval of quadrupole frequency (*ν*_Q_). In principle, the EFG reflects the distribution of electron clouds around the nucleus, which is sensitive to changes in orbital occupancy and crystal structure. As displayed in Figure 1d, the full spectrum of ^93^Nb in (LaSe)_1.14_(NbSe_2_) has multiple peaks, and the full spectrum can be well fitted by considering the quadrupole effect. Compared to the ^93^Nb NMR results for bulk 2H‐NbSe_2_, the *ν*_Q_ of (LaSe)_1.14_(NbSe_2_) is reduced to 1.3 MHz from 2.5 MHz for bulk 2H‐NbSe_2_.^[^ ^21^ ^]^ In our case, the LaSe layers provide electron doping for the NbSe_2_ layers to change the orbital occupation of ^93^Nb, which can explain the change in *ν*_Q_. The lattice misfit causes each ^93^Nb nucleus to experience different locations and local fields, resulting in a distribution of *ν*_Q_ that broadens the NMR spectrum. In our case, the relative quadrupole broadening (*δν*_Q_/*ν*_Q_) increases from 0.64% in bulk 2H‐NbSe_2_ to 9.5%, which is even larger than the corresponding value of 4% after the charge density wave (CDW) transition in bulk 2H‐NbSe_2_ (see Note S1 and Table S1, Supporting Information). This means that the homogeneity of the chemical environment of the NbSe_2_ layers in (LaSe)_1.14_(NbSe_2_) is even worse than that of bulk 2H‐NbSe_2_ after the CDW transition. Previously, it has been proven by experiments that the misfit structure only causes great modulation at the interface and has little effect on the transition metal atoms in the TX_2_ layers.^[^ ^18^ ^]^ However, according to the present results, the effect of the misfit structure is not limited to the atoms at the interface but also occurs in the entire crystal structure.

### 2.2 Anisotropic Resistivity and Hall Effect

**Figure 2:**
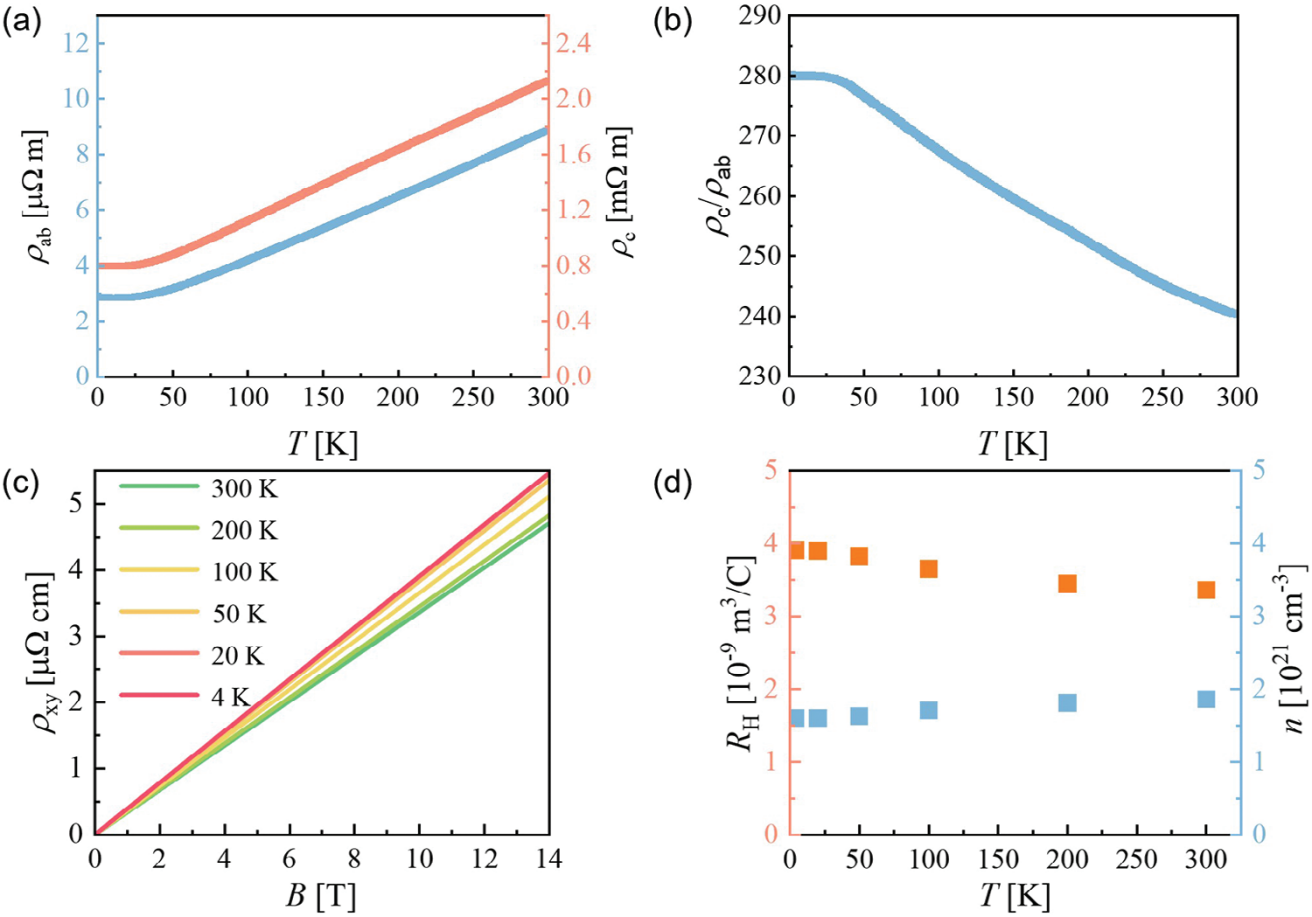
Quasi‐2D electrical transport properties of (LaSe)_1.14_(NbSe_2_). a) Temperature‐dependent *ρ_c_* and *ρ_ab_* of (LaSe)_1.14_(NbSe_2_). b) The corresponding ratio *ρ_c_*/*ρ_ab_* as a function of temperature. c) The field dependences of the Hall resistivity *ρ_xy_* at different temperatures. d) The Hall coefficient *R_H_* and carrier density *n* at different temperatures. *n* in our system is 1.86 × 10^−21^ cm^−3^ at 300 K, which is slightly lower than that reported in the literature (2 × 10^−21^ cm^−3^).^[^ ^20^ ^]^ This discrepancy is attributed to Se vacancies or slight Nb self‐doping in our sample, which is indicated by the molar ratio of La, Nb, and Se (see Note S3 and Table S2, Supporting Information). This could also explain why our sample has a slightly lower RRR (3.1) than the others (4.3).^[^ ^20^ ^]^

**Figure** **2**a illustrates the off‐plane resistivity (*ρ_c_*) and in‐plane resistivity (*ρ_ab_*) of (LaSe)_1.14_(NbSe_2_) from 300 to 4.2 K. Both the *ρ_c_* and *ρ_ab_* display metallic behavior at *T* > 30 K and gradually saturate at low temperatures. The resistivity measurements reveal that the resistance residual ratio (RRR = *R*_ab, 300 K_/*R*_ab, 4.2 K_) is 3.1 in our sample. Figure 2b shows the evolution of the anisotropic resistivity (*ρ_c_*/*ρ_ab_*) with temperature. The resistivity exhibits a large anisotropy in the range of 240–280 from 300 to 4.2 K. In high quality bulk NbSe_2_, ρ_*c*_/ρ_*ab*_ is ≈100, which is lower than that of (LaSe)_1.14_(NbSe_2_).^[^ ^22^ ^]^ The different chemical potentials of neighboring blocks induce substantial electron transfer from the LaSe layers to the NbSe_2_ layers, which makes the LaSe layers nearly insulating.^[^ ^23^ ^]^ As shown in Figure 1b,c, the LaSe layers increase the distance between adjacent NbSe_2_ layers. All of these factors make our system more 2D.

As previously mentioned, LaSe layers not only effectively reduce the dimensionality of NbSe_2_ but also induce substantial electron doping in the NbSe_2_ layers. Theoretical calculations and experiments suggest that the (LaSe)_1.14_(NbSe_2_) system can be seen as an “infinite” stack of heavily electron‐doped NbSe_2_ monolayers.^[^ ^20^ ^]^ In 2H‐NbSe_2_, the number of bands crossing the Fermi surface decreases as the number of layers decreases.^[^ ^24^ ^]^ In the bulk system, three bands cross the Fermi level. One of the bands is derived from the Se p_z_ orbital, which forms a 3D Fermi surface. Other bands originate from the Nb 4d orbitals, one of which is strictly 2D, and the other has a dispersion along the k_z_‐direction. At the monolayer limit, only one Nb 4d band with 2D characteristics remains at the Fermi level. Hence, it is anticipated that the Hall effect in (LaSe)_1.14_(NbSe_2_) will satisfy the one‐band model. Considering that the hole carrier dominates the transport properties in bulk 2H‐NbSe_2_, electron doping from LaSe layers will reduce the carrier density (*n*) of our system. The magnetic field dependences of the Hall resistance (*ρ_xy_*) at different temperatures are shown in Figure 2c. It is clear that *ρ_xy_* varies linearly with the magnetic field and that the Hall coefficients (*R_H_*) are positive in the whole temperature range, which supports that only one type of hole carrier dominates the transport properties, as we discussed above. The *R_H_* and *n* obtained by one‐band model fitting are presented in Figure 2d. Both *R_H_* and *n* only show weak temperature‐dependent behavior. In bulk 2H‐NbSe_2_, the positive *R_H_* changes sign to negative at low temperatures due to the formation of 3×3 CDW.^[^ ^25^ ^]^ Here, *R_H_* is positive at all temperatures, indicating that such a CDW transition disappears due to electron doping.^[^ ^19^ ^]^ Compared with bulk, charge transfer causes an approximately one‐order‐of‐magnitude reduction in *n* in (LaSe)_1.14_(NbSe_2_),^[^ ^25^ ^]^ which is the main reason for the decrease in *T*_c_ (1.23 K) in this system.^[^ ^20^ ^]^ (see Note S2 and Figure S1, Supporting Information).

### 2.3 Quasi‐2D Weak Antilocalization Effect from Spin‐Orbit Coupling

**Figure 3:**
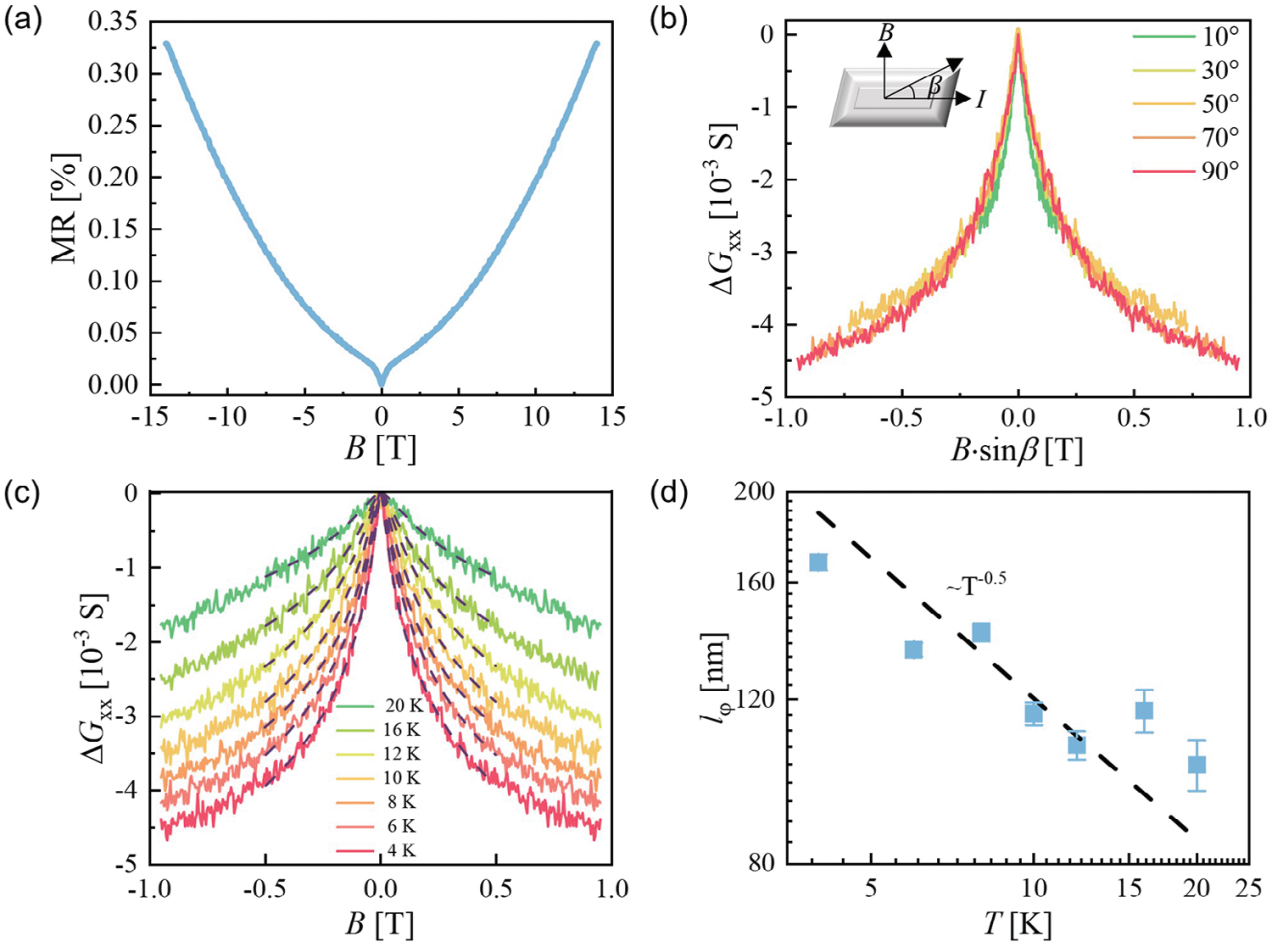
Magnetotransporter properties of (LaSe)_1.14_(NbSe_2_). a) The change in MR with the magnetic field at 4 K. b) The change in Δ*G* with a perpendicular component of the magnetic field at different angles *β*. The inset is a schematic diagram of the MR measurement device. c) The field dependences of Δ*G* at different temperatures. The dashed black lines are fitting results. d) Evolution of the phase coherence length *l*_φ_ with temperature. The dashed black line is proportional to *T* ^−1/2^.

The WAL effect is expected to appear at low temperatures in 2D metallic systems with disorder and the SOC effect, which has been observed in many 2D TMDs, such as VSe_2_ and WSe_2_.^[^ ^26^, ^27^ ^]^ As shown in Figure 1a, the SOC effect causes remarkable band splitting at the K (K') points in (LaSe)_1.14_(NbSe_2_). Moreover, the misfit structure leads to significant EFG broadening in ^93^Nb NMR, suggesting the existence of a prominent disorder effect in the NbSe_2_ layer. Therefore, we measured magnetoresistance (MR) to detect the possible WAL effect in (LaSe)_1.14_(NbSe_2_), although this effect is absent in bulk 2H‐NbSe_2_.^[^ ^25^, ^28^ ^]^ As shown in **Figure** **3**a, the transverse MR ([(ρ(H)  −  ρ(0)]/ρ(0) × 100%) is measured with the applied magnetic field parallel to the *c*‐axis and the current flowing along the *ab* plane. In the low magnetic field region, a sharp dip is clearly observed, which is a typical sign of the WAL effect. To further clarify the origin of the low‐field dip feature, the magnetoconductance Δ*G*  =  *G*(***B***) − *G*(0) at different angles (*β*) between the sample and the magnetic field was measured at 4 K. If the WAL effect originates from the 3D bulk channel, ΔG is isotropic and independent of *β*. As shown in Figure 3b, the angle‐dependent Δ*G* curves merge into a single curve at all *β* when they are plotted as a function of ***B*** · sin(β). This confirms that the WAL effect originates from 2D conduction channels in the bulk. Furthermore, we fit the field‐dependent magnetoconductance curves at different temperatures quantitatively. At the strong SOC limit, the WAL effect in a 2D system can be well described by the Hikami–Larkin–Nagaoka (HLN) equation^[^ ^29^ ^]^:

(1) $$\Delta G\;\left(B\right)=\;-\frac{\alpha e^{2}}{\pi h}\left[\psi \left(\frac{1}{2}+\frac{h}{8\pi e{l_{\varphi }}^{2}B}\right)-\ln\left(\frac{h}{8\pi e{l_{\varphi }}^{2}B}\right)\right]$$

where *ψ*, *α*, and *l*_φ_ are the digamma function, the conduction channel parameter, and the phase coherence length, respectively. In a 2D system, *α* is equal to 0.5 for a single conductive channel. Figure 3c illustrates the field‐dependent Δ*G* with the magnetic field parallel to the *c*‐axis and temperatures from 4 to 20 K. The dashed black lines are the fitting curves. The data are fitted well with the 2D HLN equation in the range of ± 0.5 T. The temperature dependence of *l*_φ_ is summarized in Figure 3d, which roughly follows a *T* ^−1/2^ behavior. Theoretically, *l*_φ_ is expected to obey a power law *l*_φ_ ≈ *T* ^p^, where the power index *p* is determined by a dephasing mechanism. Our present results suggest that 2D electron–electron interactions should be the predominant dephasing processes at low temperatures.^[^ ^30^, ^31^ ^]^ In addition, the value of *α* is found to be on the order of 10^2^, which is much larger than the theoretical value corresponding to a single conducting channel in a 2D system. This implies that a large number of conduction channels exist in the sample. A similar phenomenon has been observed for the WAL effect in some 3D systems with strong SOC, such as LuPtSb.^[^ ^32^ ^]^ However, the angular dependence of the Δ*G* and *l*_φ_ ≈ *T* ^−1/2^ dependences strongly suggest that the observed WAL effect in our system exhibits a quasi‐2D nature. In fact, the quasi‐2D nature of the electronic structure has been revealed by previous calculations and ARPES experiments in (LaSe)_1.14_(NbSe_2_).^[^ ^20^ ^]^ Therefore, the quasi‐2D WAL effect should originate from a large number of 2D conduction channels through the Fermi surface around the K (K') points of the NbSe_2_ layers.

In hexagonal TMDs monolayer, SOC will cause the band splitting at the K (K') point. The SOC of TMDs composed of heavy atoms has the more significant effect on the energy band, and the WAL effect is easier to observe. For example, for TaSe_2_, the splitting caused by the strong SOC is ≈400 meV, and this quantum transport can be detected in low‐dimensional samples.^[^ ^33^, ^34^, ^35^ ^]^ For light atoms, such as MoS_2_, the splitting caused by SOC is ≈146 meV.^[^ ^36^ ^]^ Detection of the WAL effect in such systems requires additional conditions, such as applying a gate voltage.^[^ ^37^, ^38^ ^]^ In (LaSe)_1.14_(NbSe_2_), the splitting caused by SOC is ≈150 meV, which is very close to the value of NbSe_2_ monolayer.^[^ ^20^ ^]^ However, we observe the WAL effects only in (LaSe)_1.14_(NbSe_2_), and don't find similar reports in NbSe_2_ monolayer. In TMDs, the WAL effect can be influenced by the variations in the carrier density and electron‐phonon interaction.^[^ ^34^, ^37^, ^38^ ^]^ In (LaSe)_1.14_(NbSe_2_), LaSe layers not only affect the carrier density of NbSe_2_ layers, but also regulate lattice degrees of freedom. The incommensurate structure could induce a significant disorder effect into NbSe_2_ layers. These factors make it possible to observe the WAL effect in (LaSe)_1.14_(NbSe_2_), which also proves that MLCs is a suitable system for exploring the properties of low‐dimensional TMDs.

### 2.4 Anisotropic Spin Fluctuations Induced by Spin‐Orbit Coupling

**Figure 4:**
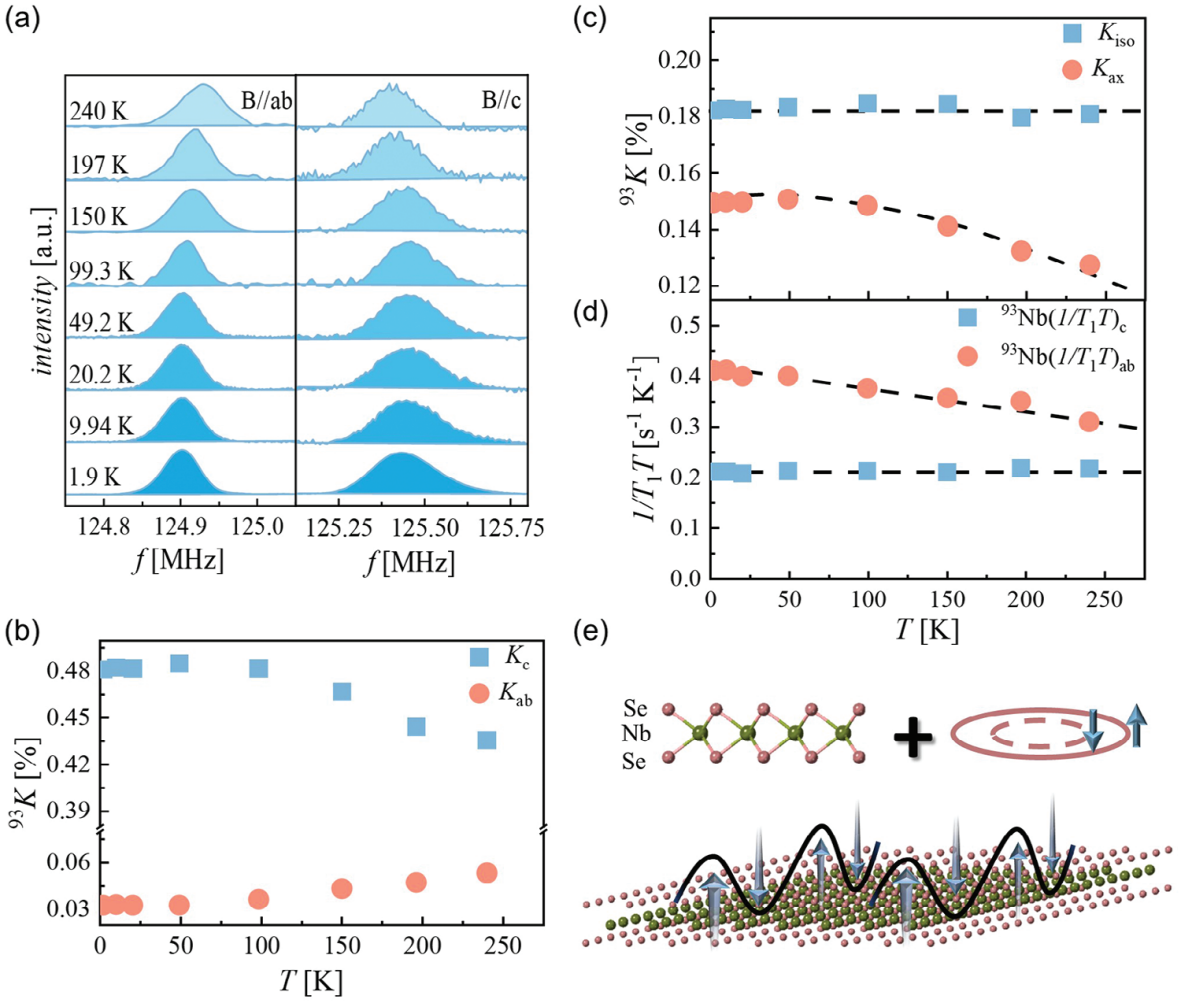
Anisotropic spin fluctuations caused by SOC in (LaSe)_1.14_(NbSe_2_). a) Line shapes of the ^93^Nb central transitions at 12 T. b) Temperature‐dependent ^93^ *K*. c) Temperature‐dependent isotropic and anisotropic ^93^ *K*. The dashed black lines are guides for eyes. d) Temperature‐dependent 1/*T*_1_ *T* of ^93^Nb. The dashed black lines are guides for eyes. e) The out‐of‐plane spin fluctuations in the noncentrosymmetric NbSe_2_ layer induced by the SOC.^[^ ^6^ ^]^

To further study the intrinsic magnetism of (LaSe)_1.14_(NbSe_2_), we measured the NMR spectra and the spin‐lattice relaxation time (*T*_1_) of the ^93^Nb central transition from 240 to 1.9 K. **Figure** **4**a shows the temperature‐dependent ^93^Nb central transition lines in (LaSe)_1.14_(NbSe_2_). Based on the temperature‐dependent NMR spectra in Figure 4a, we use the first moment to define the Knight shift (*K*) of each line with the magnetic field parallel to the *ab* plane (*K_ab_*) and the *c*‐axis (*K_c_*). In paramagnetic materials, *K* reflects the local susceptibility around the nuclei, which arises from the hyperfine interaction between the nuclei and electrons. As shown in Figure 4b, the temperature‐dependent *K* exhibits opposite trends in these two directions. Given that the NbSe_2_ layers in (LaSe)_1.14_(NbSe_2_) have a nearly undeformed 2H‐NbSe_2_ crystal structure,^[^ ^39^, ^40^ ^]^ *K* can be written as *K*  = *K_iso_*  + *K_ax_* (3cos^2^θ − 1), in which *θ* is the angle between the magnetic field and the *z*‐axis of the principal‐axis system.^[^ ^41^ ^]^ In (LaSe)_1.14_(NbSe_2_), we consider the *c*‐axis to be aligned with the *z*‐axis. In ^93^Nb, *K_iso_* represents the isotropic *K*, containing the polarization of the inner shell electron term *K_cp_* and the isotropic orbital term $K_{iso}^{orb}$. *K_ax_* comes from the dipole term *K_dip_* with the specific anisotropy and the orbital term $K_{ax}^{orb}$. Plotting *K* through this form in Figure 4c, we find that *K_iso_* remains relatively constant with temperature, which indicates that *K_cp_* and $K_{iso}^{orb}$ hardly change with temperature. Given that both *K_cp_* and *K_dip_* originate from the spin part of the electron (*K_spin_*), *K_dip_* and *K_spin_* should also be insensitive to temperature. The change in *K_ax_* with temperature is related to $K_{ax}^{orb}$, so $K_{ax}^{orb}$ and $K_{iso}^{orb}$ are obviously different with temperature. This may be due to the Ising‐type SOC in the NbSe_2_ layers, which leads to the significant anisotropy of *K_orb_*. Figure 4d shows the temperature‐dependent 1/*T*_1_ *T* with the magnetic field parallel to the *ab* plane ((1/*T*_1_ *T*)_ab_) and the *c*‐axis ((1/*T*_1_ *T*)_c_) of ^93^Nb. It can be seen that (1/*T*_1_ *T*)_ab_ increases slowly with decreasing temperature, whereas (1/*T*_1_ *T*)_c_ remains relatively constant over the whole temperature range. In general, 1/*T*_1_ *T* can be written in two parts, as 1/*T*_1_ *T* = (1/*T*_1_ *T*)_QP_  + (1/*T*_1_ *T*)_SF_. (1/*T*_1_ *T*)_QP_ comes from the quasiparticles. If only this part is considered, the change in 1/*T*_1_ *T* with temperature will be consistent with *K_spin_*, satisfying the Korringa relation.^[^ ^42^ ^]^ (1/*T*_1_ *T*)_SF_ arises from additional spin fluctuations, which increase 1/*T*_1_ *T* but have negligible effects on *K_spin_*. As we discussed above, *K_spin_* remains relatively constant with temperature, and the increase in (1/*T*_1_ *T*)_ab_ comes from additional spin fluctuations. Considering that 1/*T*_1_ *T* probes the hyperfine field perpendicular to the applied magnetic field, we believe that NbSe_2_ layers exhibit low‐energy spin fluctuations along the *c*‐axis in (LaSe)_1.14_(NbSe_2_). As shown in Figure 4e, theoretical calculations predict that SOC will induce anisotropic spin fluctuations in noncentrosymmetric TMDs.^[^ ^6^ ^]^ In (LaSe)_1.14_(NbSe_2_), the inversion symmetry of the NbSe_2_ layers is broken, and electrical transport measurements have proven that a strong SOC does exist in our system. Therefore, it is reasonable to detect spin fluctuations along the *c*‐axis in (LaSe)_1.14_(NbSe_2_), which is absent in bulk 2H‐NbSe_2_.^[^ ^43^ ^]^ Similarly, anisotropic spin fluctuations due to strong SOC have been discussed in other systems, such as Sr_2_RuO_4_.^[^ ^44^, ^45^ ^]^ In addition, we measured the *T*_1_ of ^139^La. The (1/*T*_1_ *T*)_ab_ of ^139^La is ≈1.5 × 10^−3^ s^−1^ K^−1^ at 2 K, which is much lower than the 0.4 s^−1^ K^−1^ of ^93^Nb. This indicates that most of the conduction electrons at the Fermi surface originate from ^93^Nb.

## 3 Conclusion

In conclusion, we investigated the properties of (LaSe)_1.14_(NbSe_2_) in the normal state by NMR experiments and electrical transport measurements. The physical properties of the system are dominated by NbSe_2_ layers with heavy electron doping. As the electron donor, the LaSe layers affect the microscopic electron state of the ^93^Nb nuclei in the NbSe_2_ layers. In our system, the SOC effects become apparent due to the reduction in the effective dimensionality of the NbSe_2_ layers. We have not only directly observed the quantum transport phenomena caused by SOC but also observed the anisotropic spin fluctuations from SOC in NbSe_2_ layers through NMR experiments. Therefore, MLCs derived from NbSe_2_ provide an ideal platform for studying 2D NbSe_2_. The superconductivity of 2D NbSe_2_ is predicted to exhibit fascinating physical phenomena, such as topological superconductivity and singlet‐triplet mixing of the order parameter.^[^ ^7^, ^46^, ^47^ ^]^ It is anticipated that experiments exploring the superconducting properties of MCLs will be interesting.

The large tunability of the parameter space leads to many exotic states in 2D TMDs. However, the practical application of 2D samples is dramatically limited by the difficulty of preparation and the poor stability of the films. Combined with our experiments, MLCs provide a new route to realize or study the properties of low‐dimensional materials with high stability. In fact, many bulk van der Waals materials have their own derived MLCs. For example, NbS_2_ and TaS_2_ have their own derived MLCs, such as (LaS)_1.14_(NbS_2_) and (PbS)_1.13_(TaS_2_).^[^ ^48^, ^49^ ^]^ Due to the diversity of the MX layers, the carrier density and band structure of MLCs can be tuned over a wide range. Therefore, designing and growing MLCs with superior performance is a potential area of development.

Finally, the physical properties of the MLCs are still influenced by the MX layers in some way. Different blocks have differences in chemical potential and band structure, which leads to charge transfer and even energy band reconstruction, resulting in special properties that cannot be found in single‐phase samples. We have shown that the LaSe layers have a significant effect on the microscopic electronic states of the NbSe_2_ layers. Other experiments have demonstrated that similar to 2D moiré materials, new quasi‐1D electron states will appear in MLCs along the misfit direction.^[^ ^18^ ^]^ These new electron states suggest that the discovery and study of the physical properties of MLCs is also a promising area.

## 4 Experimental Section

### Sample Preparation and Characterization

A high‐quality single crystal (LaSe)_1.14_(NbSe_2_) was grown by chemical vapor transport using I_2_ as the transport agent. First, La (99.9%), Nb (99.99%), and Se (99.999%) precursors were mixed and ground at a molar ratio of 1.14:1.0:3.14, in which millimeter‐sized fresh La powders were scraped from high‐purity La ingots in an argon atmosphere. The mixture was transferred to a 20 cm long quartz tube, which was evacuated to create a vacuum seal. Then, the quartz tube was placed in a two‐zone furnace, and the source and growth zones were set at 1000° and 900°, respectively, for 2 weeks. The single crystals were obtained by turning off the power and furnace‐cooling to room temperature. Then, the samples were washed with ethanol to remove the I_2_ on the surface. The typical dimensionality of the samples is ≈2 × 3 × 0.01mm^3^.

The good crystallinity of the samples was confirmed by XRD at room temperature. Here, a Rigaku SmartLab‐9 powder diffractometer with Cu Kα radiation was used to collect the XRD data. The chemical composition of (LaSe)_1.14_(NbSe_2_) was determined by energy‐dispersive X‐ray spectroscopy (GeminiSEM, 500). The electrical transport properties of high‐quality single crystals were measured using a Quantum Design Physical Property Measurement System (PPMS). *ρ*_c_ was measured for the Corbino shape‐like configuration with the four‐electrode method.^[^ ^50^ ^]^ The sample was mounted on the rotary platform to measure the MR with the standard four‐probe method, in which the ac‐transport option of the PPMS was used to measure MR.

### Nuclear Magnetic Resonance

A highly uniform 12 T magnetic field was provided by a magnet from Oxford Instruments, and a commercial spectrometer from Thamway was used in this study. Under an external magnetic field of 12 T, NMR experiments were performed on ^93^Nb nuclei (*I* = 9/2) and ^139^La nuclei (*I* = 7/2). The single crystal was loaded into an NMR coil made from Cu. The NMR signal generated by the ^63^Cu of the coil was used to calibrate the external magnetic field. In the experiment, the NMR spectra and *T_1_* were measured with the magnetic field parallel and perpendicular to the *c*‐axis, respectively. Under a fixed magnetic field, the spectra of ^93^Nb were obtained by scanning the *rf* frequency and integrating the spin echo. The *T*_1_ values of the central transitions were measured by the standard inverse recovery method at all temperatures. The fitting of the NMR spectrum was explained in the Supporting Information.

### First‐Principles Calculations

The Vienna ab initio simulation package (VASP) was employed to calculate the band structure.^[^ ^51^ ^]^ The Perdew–Burke–Ernzerh (PBE) function describes the exchange correlation energy.^[^ ^52^ ^]^ The cutoff energy of the plane‐wave basis was set to 450 eV. The BZ was sampled using an 18 × 18 × 1 Monkhorst–Pack grid for NbSe_2_ monolayers. The vacuum layer was defined as 15 Å to avoid interlayer coupling between neighboring slabs. The crystal structures were fully relaxed by minimizing the forces of each atom smaller than 0.01 eV Å^−1^, and the energy convergence was set to 1.0×10^−7^ eV.

## Conflict of Interest

The authors declare no conflict of interest.

## Supporting information

Supporting Information

## Data Availability

The data that support the findings of this study are available from the corresponding author upon reasonable request.
