# Supplementary material for: Anisotropic Spin Fluctuations Induced by Spin‐Orbit Coupling in a Misfit Layer Compound (LaSe)1.14(NbSe2)
Source: Adv Sci (Weinh). 2024 Aug 29;11(40):2403824. doi: 10.1002/advs.202403824 (PMC11515895; doi:10.1002/advs.202403824)
Supplement: Supplementary file 1 — Supporting Information [file ADVS-11-2403824-s001.pdf]

ADVANCED SCIENCE
Open Access

Supporting Information

for *Adv. Sci.*, DOI 10.1002/advs.202403824

Anisotropic Spin Fluctuations Induced by Spin-Orbit Coupling in a Misfit Layer Compound $(LaSe)_{1.14}(NbSe_2)$

Min Shan, Shunjiao Li, Ye Yang, Dan Zhao, Jian Li, Linpeng Nie, Zhimian Wu, Yanbing Zhou, Lixuan Zheng, Baolei Kang, Tao Wu and Xianhui Chen**

Supporting Information

Anisotropic Spin Fluctuations Induced by Spin-orbit Coupling in a Misfit Layer Compound $(LaSe)_{1.14}(NbSe_2)$

Min Shan, Shunjiao Li, Ye Yang, Dan Zhao, Jian Li, Linpeng Nie, Zhimian Wu, Yanbing Zhou, Lixuan Zheng, Baolei Kang, Tao Wu and Xianhui Chen**

Supplementary Note 1: Fitting the NMR Spectra of Bulk 2H-$NbSe_2$ and $(LaSe)_{1.14}(NbSe_2)$

We compare the ^{93}Nb NMR results of bulk 2H-$NbSe_2$ and $(LaSe)_{1.14}(NbSe_2)$ at different temperatures with the magnetic field along the *c*-axis, in which the spectra of bulk 2H-$NbSe_2$ are taken from the literature. [1] When the Zeeman energy is much greater than the quadrupole energy in the experiment, according to the second-order perturbation theory, the frequency of ^{93}Nb is given by: [2]

$$f = \gamma H_0(1+K) + \frac{\sqrt{6}eQ}{432\hbar}\left(3m^2 - \frac{99}{4}\right)V_0 - \frac{1}{\gamma_N H_0(1+K)}\left(\frac{eQ}{144\hbar}\right)^2$$

$$\times\left[2V_{-1}V_1 m(98-8m^2) + 2V_{-2}V_2 m\left(\frac{97}{2} - 2m^2\right)\right]$$

$$K = K_x\sin^2\theta\cos^2\varphi + K_y\sin^2\theta\sin^2\varphi + K_z\cos^2\theta$$

$$V_0 = \sqrt{\frac{3}{2}}eq[\frac{1}{2}(3\cos^2\theta - 1) + \frac{1}{2}\eta\sin^2\theta\cos2\varphi]$$

$$2V_{-1}V_1 = -\frac{3}{2}e^2q^2\left[\left(-\frac{1}{3}\eta^2\cos^2 2\varphi + 2\eta\cos2\varphi - 3\right)\cos^4\theta\right.$$

$$\left. + \left(\frac{2}{3}\eta^2\cos^2 2\varphi - 2\eta\cos2\varphi - \frac{1}{3}\eta^2 + 3\right)\cos^2\theta + \frac{1}{3}\eta^2(1-\cos^2 2\varphi)\right]$$

$$V_{-1}V_1 = \frac{3}{2}e^2q^2\left[\left(\frac{1}{24}\eta^2\cos^2 2\varphi - \frac{1}{4}\eta\cos2\varphi - \frac{3}{8}\right)\cos^4\theta + \left(-\frac{1}{12}\eta^2\cos^2 2\varphi + \frac{1}{6}\eta^2 - \frac{3}{4}\right)\cos^2\theta + \frac{1}{24}\eta^2\cos^2 2\varphi + \frac{1}{4}\eta\cos2\varphi + \frac{3}{8}\right]$$

$$\upsilon_Q = 3e^2q^2/72\hbar$$

where θ and φ are the Euler angles between field H_0 and the principal axis of the electric field gradient (EFG), γ is the gyromagnetic ratio, *m* is the magnetic quantum number, and η is the asymmetry parameter. Considering the inhomogeneity of the chemical environment in the system, we used a normal distribution for *K* and ν_Q with standard deviations of δK and $\delta\nu_Q$, respectively. Considering that the $NbSe_2$ layers in $(LaSe)_{1.14}(NbSe_2)$ have a nearly undeformed 2H-$NbSe_2$ structure, we selected an η equal to zero during the fitting process. The fitting results are shown in Table S1.

Table S1. Fitting parameters for the ^{93}Nb NMR spectra of bulk 2H-$NbSe_2$ and $(LaSe)_{1.14}(NbSe_2)$.

	$(LaSe)_{1.14}(NbSe_2)_{(100\ K)}$	$NbSe_{2(100\ K)}$	$NbSe_{2(10\ K)}$
v_Q [Mhz]	1.3	2.5	2.53
δv_Q [Mhz]	0.12	0.016	0.1
$\delta v_Q/v_Q$	9.2%	0.64%	4%
K [%]	0.48%	0.38%[3]	0.37%[3]
δK	0.06%	0.065%	0.1%
$\delta K/K$	15%	17%	27%

Supplementary Note 2: The Change in T_c with Carrier Density

Figure S1 summarizes the T_c and carrier density (n) at 50 K of bulk 2H-$NbSe_2$ and $(LaSe)_{1.14}(NbSe2)_{n=1,2}$. We found that similar to field-effect transistor (FET) gating, [4] as electron doping increases, n decreases, which causes T_c to decrease.

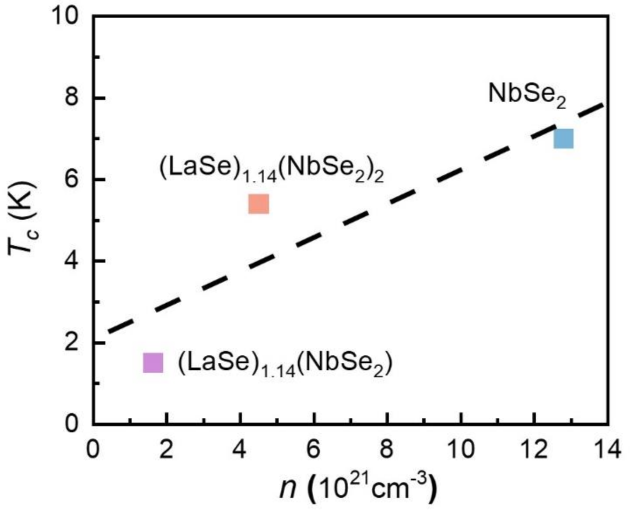

Figure S1. The relationship between T_c and n at 50 K. The data for $(LaSe)_{1.14}(NbSe_2)_2$ and bulk 2H-$NbSe_2$ come from the literature. [5,6] The dashed black lines are visual guides.

Supplementary Note 3: Sample Characterization with EDS

Table S2. The molar ratio of La, Nb and Se determined by EDS.

	La	Nb	Se
Sample 1	19.78%	20.64%	59.58%
Sample 2	19.75%	20.51%	59.74%
Sample 3	19.94%	20.5%	59.56%
Sample 4	19.77%	20.64%	59.59%
Sample 5	19.64%	20.89%	59.47%
Average	19.776%	20.636%	58.588%
Average	1.14	1.19	3.38

As shown in Table S2, the chemical element molar ratio of $(LaSe)_{1.14}(NbSe_2)$ was obtained by energy-dispersive X-ray spectroscopy (EDS). Similar to the literature, [6] our sample also contains La vacancies, which can compensate for electron doping in the $NbSe_2$ layers. We note that the n of our system (1.86×10^{-21} cm^{-3}) is slightly lower than that reported in the literature (2×10^{-21} cm^{-3}) at 300 K. This is due to the Se vacancies or the slight Nb self-doping in our system within the accuracy error range of EDS.

Supplementary Note 4: Quadrupole Shift in Knight Shift

In our case, the first moment of the spectrum is used to define the average Knight shift. In general, in addition to the magnetic shift, the quadrupole shift also contributes to the Knight shift of the center transition. As shown in Figure S2, we measured the spectra at 1.9 K and 150 K, and the extracted quadrupole frequency showed almost no temperature dependence. Therefore, the quadrupole shift only contributes to a temperature-independent shift, which does not affect the temperature-dependent relationship of the averaged Knight shift.

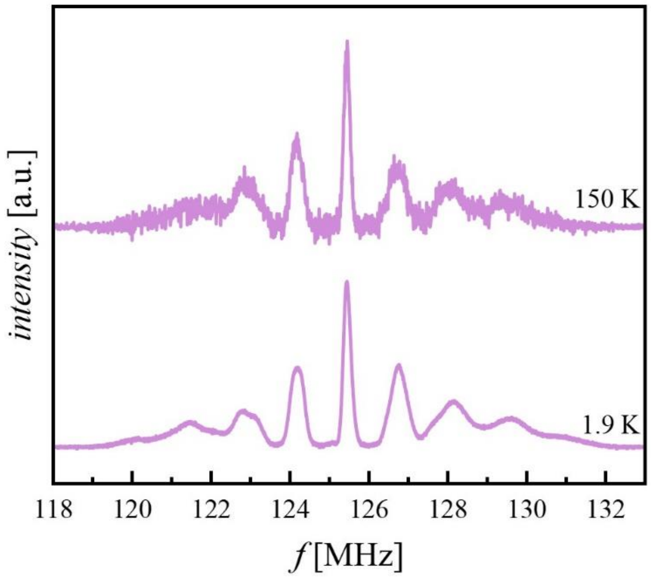

Figure S2. ^{93}Nb NMR spectra at 1.9 K and 150 K with a magnetic field applied along the *c*-axis.

References

[1] D. M. Wilson, *Doctor Thesis,* Florida State University **2017**.
[2] P. P. Man, In *Encyclopedia of Analytical Chemistry*, a6111, (Ed: R. A. Meyers), John Wiley & Sons, Ltd, Chichester, UK, 2006, https://onlinelibrary.wiley.com/doi/10.1002/9780470027318.a6111.
[3] K. Ghoshray, B. Pahari, A. Ghoshray, V. V. Eremenko, V. A. Sirenko, B. H. Suits, *J. Phys.: Condens. Matter*. **2009**, 21, 155701.
[4] X. Xi, H. Berger, L. Forró, J. Shan, K. F. Mak, *Phys. Rev. Lett*. **2016**, 117, 106801.
[5] L. J. Li, Z. A. Xu, J. Q. Shen, L. M. Qiu, Z. H. Gan, *J. Phys.: Condens. Matter.* **2005**, 17, 493.
[6] T. Samuely, D. Wickramaratne, M. Gmitra, T. Jaouen, O. Šofranko, D. Volavka, M. Kuzmiak, J. Haniš, P. Szabó, C. Monney, G. Kremer, P. L. Fěvre, F. Bertran, T. Cren, S. Sasaki, L. Cario, M. Calandra, I. I. Mazin, P. Samuely, *Phys. Rev. B* **2023**, 108, L220501.
